# IL-10 Counteracts Proinflammatory Mediator Evoked Oxidative Stress in Caco-2 Cells

**DOI:** 10.1155/2014/982639

**Published:** 2014-07-23

**Authors:** Eva Latorre, Nyurky Matheus, Elena Layunta, Ana Isabel Alcalde, José Emilio Mesonero

**Affiliations:** ^1^Department of Pharmacology and Physiology, Faculty of Veterinary Sciences, University of Zaragoza, Miguel Servet 177, 50013 Zaragoza, Spain; ^2^Department of Basic Sciences, Faculty of Veterinary Sciences, University of Centroccidental Lisandro Alvarado, Núcleo Hector Ochoa Zuleta, Tarabana 3023, Lara, Venezuela

## Abstract

Oxidative stress is thought to play a key role in the development of intestinal damage in intestinal inflammatory diseases. Several molecules are involved in the intestinal inflammation, either as pro- or anti-inflammatory factors; however, their effects on intestinal oxidative stress seem to be controversial. This work analyzes the contribution of pro- and anti-inflammatory molecules to the balance of oxidative damage in intestinal epithelial cells, as well as their effects on cellular antioxidant enzyme activity. With this purpose, the lipid and protein oxidation, together with the activity of catalase, superoxide dismutase, and glutathione peroxidase, were determined in the Caco-2 cells treated with serotonin, adenosine, melatonin, and TNF*α*, as proinflammatory factors, and IL-10, as an anti-inflammatory cytokine. The results have shown that all the proinflammatory factors assayed increased oxidative damage. In addition, these factors also inhibited the activity of antioxidant enzymes in the cells, except melatonin. In contrast, IL-10 did not alter these parameters but was able to reduce the prooxidant effects yielded by serotonin, adenosine, melatonin, or TNF*α*, in part by restoring the antioxidant enzymes activities. In summary, proinflammatory factors may induce oxidative damage in intestinal epithelial cells, whereas IL-10 seems to be able to restore the altered redox equilibrium in Caco-2 cells.

## 1. Introduction

Intestinal inflammation is an adaptive response that is triggered by adverse conditions such as tissue injury and infection. Moreover, intestinal inflammation alters the efficiency and the whole physiology of the intestinal tract. The integrity of the epithelial barrier is essential for preventing the development of inflammatory conditions in the intestine; in fact, it is well known that the structure and the function of intestinal barrier are compromised in inflammatory bowel diseases (IBDs). The specific pathways leading to cellular damage in chronic intestinal inflammation are not completely understood; however, oxidative stress is a potential triggering factor for IBDs [1]. In this context, several proinflammatory mediators may generate further oxidation products, leading to a self-sustaining and autoamplifying vicious circle that eventually impairs the gut barrier thus aggravating inflammatory damage [2]. This kind of process has been also described in the cardiovascular system [3].

Several gastrointestinal (GI) molecules that modulate GI activity [4–6] have also been noted as being involved in intestinal inflammation. Thus, some of these molecules at high concentrations may act as proinflammatory factors. This is the case for serotonin (5-HT) [7, 8], adenosine [9, 10], and TNF*α* [11], whereas others, such as melatonin, may act either as a proinflammatory factor at high concentrations or as an anti-inflammatory molecule depending on the cell conditions [12]. Moreover, interleukin-10 (IL-10), a well-known anti-inflammatory cytokine [13–15], has also been shown to modulate intestinal physiology [16].

In the last decade, the incidence of intestinal inflammatory diseases has been growing, and there is an increasing interest in understanding the mechanisms by which physiological GI molecules, present in the intestinal mucosa, may contribute to intestinal inflammation by affecting intestinal epithelium. Thus, the aim of the present work has been the analysis of the effect of 5-HT, adenosine, melatonin, and TNF*α*, acting as proinflammatory factors, and IL-10, acting as an anti-inflammatory cytokine, in the intestinal epithelial cells' redox equilibrium.

## 2. Materials and Methods

### 2.1. Reagents

The following drugs and substances were used (abbreviations and suppliers in parentheses): serotonin (5-HT), melatonin, adenosine, glutathione reductase, L-glutathione reduced, *β*-nicotinamide adenine dinucleotide phosphate (NADPH), tert-butyl hydroperoxide, 1-methyl-2-phenylindole, acetonitrile, methane sulfonic acid, 1,1,3,3-tetramethoxypropane, 2,4-dinitrophenylhydrazine (DNPH), trichloroacetic acid, guanidine, xanthine, xanthine oxidase (Sigma-Aldrich, St. Louis, MO, USA), TNF*α*, and IL-10 (Peprotech, Rocky Hill, NJ, USA). All generic reagents were purchased from Sigma-Aldrich and Roche Applied Sciences (Sant Cugat del Vallés, Barcelona, Spain).

### 2.2. Cell Culture and Cell Homogenate Preparation

Caco-2/TC7 cells have been used in the present study since they are an excellent human enterocyte-like model to study intestinal epithelial physiology [17, 18]. The cells were always used between passages 19 and 35 and were cultured at 37°C in an atmosphere of 5% CO_2_ and maintained in high-glucose DMEM supplemented with 2 mM glutamine, 100 U/mL penicillin, 100 *μ*g/mL streptomycin, 1% nonessential amino acids, and 20% heat-inactivated fetal bovine serum (FBS) (Life Technologies, Carlsbad, CA). The cells were passaged enzymatically (0.25% trypsin-1 mM EDTA), subcultured in 25 cm^2^ plastic culture flasks (Sarstedt, Nuembrecht, Germany), and seeded at a density of 10^4^ cells/cm^2^. The medium was changed 48 h after seeding and daily thereafter. The experiments were carried out in the cells 14 days after seeding (nine days after confluence), and the cell medium was FBS-free for 24 h before using the cells. Cell activity through different passages and after a postconfluence condition has been shown to be normal [18]. For the experiments, cells were seeded in 6-well plates at a density of 2 × 10^5^ cells/well. Two wells per condition (treatment) were used in each independent experiment, which corresponded to a different passage of Caco-2/TC7 cells. The measurement of the different parameters was performed in triplicate for each well cell culture condition. Serotonin 100 *μ*M, adenosine 100 *μ*M, melatonin 5 mM, TNF*α* 5 ng/mL, and/or IL-10 at two concentrations (0.01 and 25 ng/mL) were added to the cell medium for one day before the measurement of oxidative parameters. Cell status under the treatments was assessed by both microscopy and cell count, and none of the treatments appeared to affect cell culture morphology, cell proliferation, or cell viability (data not shown).

For cell homogenate preparation, the cells were resuspended and homogenized with a cold Tris-manitol buffer (Tris 2 mM, mannitol 50 mM, pH 7.1, protease inhibitors, and 0.02% sodium azide). Then, the homogenate was disrupted by sonication (15 1-s bursts, 60 W). Antioxidant enzymes' activity was measured in the cellular homogenate. For lipid peroxidation and protein carbonyl analysis, the homogenate was additionally centrifuged for 10 min at 3,000 g, and the supernatant was taken for the study. Protein content was measured by following the Bradford method (Bio-Rad, Hercules, CA, USA).

### 2.3. Measurement of Lipid and Protein Peroxidation

The level of lipid peroxidation was determined by measuring the concentration of malondialdehyde (MDA) and 4-hydroxyalkenals (4-HDA), as described previously [19]. Briefly, MDA+4-HDA reacted with N-methyl-2-phenylindole and yielded a stable chromophore that was measured in a spectrophotometer at 586 nm, using 1,1,3,3-tetramethoxypropane as standard. The results were calculated in nmol MDA+4-HDA/mg protein and were expressed as the percentage of the control value (100%).

Protein oxidation was analyzed by carbonyl level measurement as previously described [19]. Cell homogenates were incubated with the classical carbonyl reagent DNPH, and protein carbonylation was measured spectrophotometrically at 375 nm. The results were calculated in nmol carbonyl groups/mg protein and were expressed as percentage of the control value (100%).

### 2.4. Analysis of Antioxidant Enzymes' Activity

The activity of the antioxidant enzymes catalase (CAT), superoxide dismutase (SOD), and glutathione peroxidase (GPx) was measured by adding specific substrates and measuring the rates of disappearance of the substrates, following protocols previously described [19]. CAT activity was calculated as the reduction of H_2_O_2_ in nmol/mg protein × min that was measured spectrophotometrically at 240 nm.

In relation to SOD, the cytosolic superoxide dismutase CuZnSOD form was analyzed. SOD activity was measured by the production of superoxide radicals that was determined with a xanthine/xanthine-oxidase system, which induces a cytochrome C reduction. This reduction was measured at 550 nm and SOD activity calculated as U/mg prot × min.

The GPx analyzed in our study was the GPx2 form (GPx-GI), which is cytoplasmatic and the most abundantly expressed in GI tract and liver. GPx activity was measured in a cell homogenate mixed with potassium phosphate, pH 7.0, containing 10 mM glutathione, 0.25 units of glutathione reductase, and 1.5 mM *β*-NADPH. The decrease in NADPH during oxidation of NADPH to NADP is indicative of GPx activity, which was measured spectrophotometrically at 340 nm and calculated as NADPH nmol/mg prot × min. The results were expressed as percentages of CAT, SOD, and GPx activities of control value (100%).

### 2.5. Statistical Analysis

All results are expressed as means ± the standard error of the mean (SE) of five independent experiments. Statistical comparisons were performed using one-way ANOVA followed by the Bonferroni post-test with a confidence interval of 95% (*P* < 0.05). Normal distribution was previously confirmed with the D'Agostino-Pearson test. Statistical analysis was carried out using the computer-assisted Prism GraphPad Program (Prism version 4.0, GraphPad Software, San Diego, CA).

## 3. Results

### 3.1. Effect of 5-HT, Adenosine, Melatonin, TNF*α*, and IL-10 on Lipid and Protein Oxidation in Caco-2 Cells

**Figure 1:**
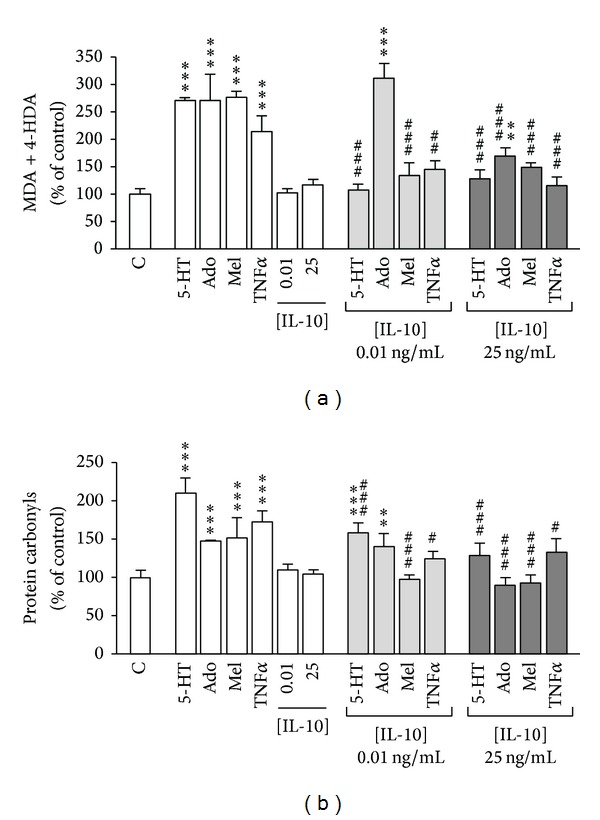
Effect of 5-HT, adenosine, melatonin, TNF*α*, and/or IL-10 on lipid peroxidation (a) and protein oxidation (b). Caco-2 cells were treated throughout the course of one day with either 5-HT 100 *μ*M, adenosine (Ado) 100 *μ*M, melatonin (Mel) 5 mM, TNF*α* 5 ng/mL, or IL-10 at two concentrations, 0.01 or 25 ng/mL. Results were expressed as the percentages of the control value (100%) and were indicated as the mean ± SE of five independent experiments. **P < 0.01, and ***P < 0.001, compared with control (C); ^#^ *P* < 0.05, ^##^ *P* < 0.01, and ^###^ *P* < 0.001 compared with the corresponding condition of treatment without IL-10.

The results show that Caco-2 cells treated with 5-HT, adenosine, melatonin, or TNF*α* significantly increased lipid peroxidation (Figure 1(a)) and protein carbonyl (Figure 1(b)) levels. Conversely, IL-10 did not appear to modify the cell oxidative status. However, IL-10, at the two concentrations assayed, was able to reduced lipid and protein oxidation induced by 5-HT, melatonin and TNF*α*. Oxidative damage in lipids and proteins yielded by adenosine was only reverted by IL-10 at high concentration (25 ng/mL) (Figure 1).

### 3.2. Analysis of the Activity of Antioxidant Enzymes CAT, SOD and GPx in Caco-2 Cells Treated with 5-HT, Adenosine, Melatonin, TNF*α* and IL-10

**Figure 2:**
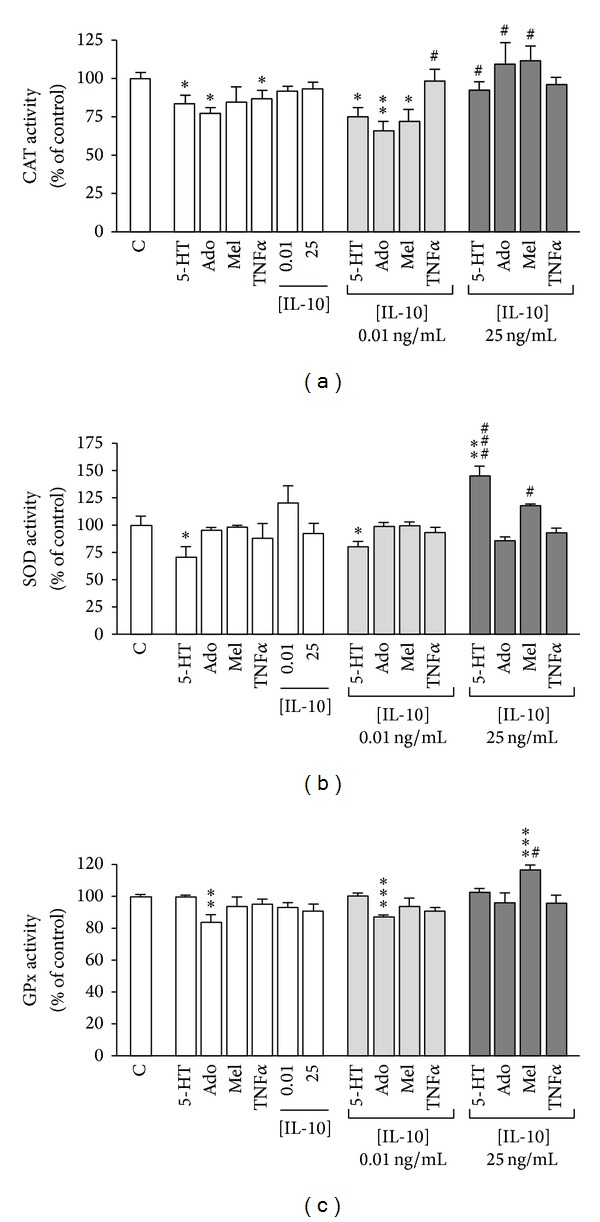
Effect of 5-HT, adenosine, melatonin, TNF*α*, and/or IL-10 on CAT (a), SOD (b), and GPx (c) activity. Caco-2 cells were treated throughout the course of one day with 5-HT 100 *μ*M, adenosine (Ado) 100 *μ*M, melatonin (Mel) 5 mM, TNF*α* 5 ng/mL, or IL-10 at two concentrations, 0.01 or 25 ng/mL. Results were expressed as percentages of the enzyme activity of control cells (100%) and were indicated as the mean ± SE of five independent experiments. *P < 0.05, **P < 0.01, and ***P < 0.001, compared with control (C); ^#^ *P* < 0.05, and ^###^ *P* < 0.001 compared with the corresponding condition of treatment without IL-10.

The results have shown that 5-HT, adenosine, and TNF*α* significantly reduced CAT activity, which did not seem to be significantly affected by melatonin or IL-10 at the two concentrations assayed. In combined treatments, IL-10 at a low concentration (0.01 ng/mL) seemed to reverse CAT inhibition yielded by TNF*α* and, on the contrary, it showed to increase the inhibition of CAT activity induced by 5-HT, adenosine, or melatonin. However, IL-10 at a high concentration (25 ng/mL) was able to reverse CAT inhibition induced by 5-HT and adenosine and to increase significantly CAT activity in presence of melatonin (Figure 2(a)).

In relation with SOD, 5-HT showed to reduce its activity, and this effect was not only reversed by IL-10 at a high concentration but even surpassed it (Figure 2(b)). Adenosine, melatonin, TNF*α*, and IL-10 at the two concentrations assayed did not seem to affect SOD activity. Surprisingly, treatment with IL-10 at a high concentration plus melatonin yielded a significant increase of SOD activity (Figure 2(b)).

The analysis of GPx showed that only adenosine significantly reduced GPx activity and that this effect was also reversed by IL-10 at a high concentration (Figure 2(c)). Curiously, the treatment with melatonin, which alone did not affect GPx activity, together with IL-10 at a high concentration showed an increase in antioxidant GPx activity above the control (Figure 2(c)).

## 4. Discussion and Conclusions

The results obtained in this work demonstrate that the intestinal proinflammatory treatments analyzed—5-HT, adenosine, TNF*α*, and melatonin at high concentrations—induced oxidative effects in lipids and proteins of intestinal epithelial cells. This oxidative damage did not seem to significantly affect the survival of the cells; however, it may be added to the prooxidant status settled in the intestinal cells under inflammatory conditions. The prooxidant effects of these molecules have also been noted in different tissues [11, 20, 21]. In contrast, the anti-inflammatory cytokine IL-10, which did not appear to affect lipid and protein oxidation at any concentration assayed, was shown to reverse the oxidative damage in lipids and proteins induced by the proinflammatory molecules tested, except IL-10 a low concentration, which did not significantly reduce the lipid peroxidation and protein carbonyl levels yielded by adenosine. These results suggest that IL-10 effects may be evident in tissues that have been exposed to inflammatory conditions, as a previous study has concluded [22].

Antioxidant enzyme activity is the main way to restore redox equilibrium in cells, and intestinal inflammation has been characterized by marked but differing alterations of various enzymes involved in oxidant/antioxidant intestinal epithelium homeostasis. In terms of intestinal inflammatory diseases, SOD [23, 24] and CAT activities [25, 26] have been observed to be altered in the intestinal mucosa of IBD; however, the results of GPx activity have been shown to be more controversial [27, 28]. In this context, CAT, SOD, and GPx activities were measured in order to analyze whether they were involved in both the oxidative effects yielded by proinflammatory molecules and the cell oxidative equilibrium restoration carried out by IL-10.

The proinflammatory factors tested have been shown to significantly reduce the activity of some of the antioxidant enzymes, even though this effect did not appear to be the only responsible for their observed prooxidant action on lipids and proteins. Thus, our results suggest that the oxidative effect of 5-HT in Caco-2 cells may be in part due to the inhibition of CAT and SOD activity. In agreement with these results, a previous study made in cardiac myocytes has demonstrated that 5-HT increased the level of H_2_O_2_ [29]. In the case of adenosine, it has been shown to reduce CAT and GPx activity, which may contribute to its effect on the oxidative damage of lipids and proteins. Adenosine effects on antioxidant enzymes have not been deeply analyzed; despite this, the activity of adenosine deaminase and its negative correlation with CAT, SOD, and GPx activities has been demonstrated in plasma and blood cells [30]. In relation to TNF*α*, its prooxidant effect might be in part mediated by the inhibition of CAT activity, as it has been recently described in osteoblasts [31]. Finally, in contrast, melatonin did not appear to affect any enzyme activity measured, and consequently, these antioxidant enzymes did not seem to be involved in the strong oxidative stress yielded by melatonin in proteins and lipids. Melatonin is considered an antioxidant molecule, but it may also act as a prooxidant factor. In fact, melatonin has been shown to generate reactive oxygen species [12]. Moreover, and in agreement with our results, it has been previously suggested that melatonin may induce an indirect prooxidant effect that contributes to acute inflammation [32].

In relation to IL-10, this cytokine alone did not appear to alter the antioxidant enzymes activities analyzed at any of the two concentrations assayed, which may explain its lack of effect on lipid and protein oxidation in Caco-2 cells. In contrast, under co-treatment with the proinflammatory mediators IL-10 at a high concentration was shown to reverse their inhibitory effect on the different antioxidant enzymes activities. Thus, IL-10 at a high concentration reversed CAT and SOD inhibition induced by 5-HT, and CAT and GPx inhibition yielded by adenosine. In contrast, IL-10 at a low concentration did not seem to reverse any effect on SOD and GPx activity inhibition, but surprisingly it seemed to restore CAT activity inhibited by TNF*α* treatment. In agreement with these results, recent studies have demonstrated the ability of IL-10 to reduce H_2_O_2_ production induced by IFN-*γ* and TNF*α*-activated macrophages [33]. Surprisingly, IL-10 at low concentration seemed to aggravate CAT activity inhibition induced by 5-HT, adenosine, and melatonin.

From our results, it can be inferred that IL-10 effects depend on its concentration, as it has been recently described in Caco-2 cells [16], and in cardiovascular events [34]. The protective effect of IL-10 against the oxidative damage induced by prooxidant molecules has also been demonstrated in cardiomyocytes [35]. Recent results in endothelium have suggested that the antioxidant effect of IL-10 under proinflammatory conditions may be explained by the activation of PI3k signaling [36]. Since this intracellular pathway has also been recently described in Caco-2 cells [16] as triggered by IL-10, it may be inferred that PI3k pathway might also mediate IL-10 antioxidant effects in intestinal epithelial cells.

Interestingly, CAT, SOD, and GPx activities, which remained unaltered under treatment with either melatonin or with IL-10 at high concentrations, were significantly augmented by the cotreatment of the cells with both molecules, thus suggesting a synergistic antioxidant action of both factors. Our results agree with previous studies that concluded that melatonin may exert a beneficial effect in the inflammatory processes by stimulating IL-10 production (reviewed in [37, 38]).

In brief, our study demonstrates that GI molecules involved in intestinal inflammation may also act as prooxidant factors in intestinal epithelial cells. This effect may be due in part to the inhibition of the activity of antioxidant enzymes. This prooxidant effect might contribute to a worsening of inflammation by damaging the intestinal epithelium. In addition, IL-10, mainly at high concentrations, may reverse the prooxidative effects induced by the proinflammatory factors, thus suggesting that IL-10 may contribute to the improvement of the inflammation not only through anti-inflammatory but also through antioxidant effects. This antioxidant effect of IL-10 in the intestinal epithelium reinforces the role of this cytokine as a successful therapy to treat intestinal inflammation and IBDs.

The results of the current study may be useful for clarifying the processes involved in intestinal inflammation and may contribute to the design of specific therapies in the treatment of intestinal inflammatory diseases by acting on the redox balance.
